# Maintaining effective mass drug administration for lymphatic filariasis through in-process monitoring in Sierra Leone

**DOI:** 10.1186/1756-3305-5-232

**Published:** 2012-10-12

**Authors:** Mary H Hodges, Mustapha Sonnie, Hamid Turay, Abdulai Conteh, Florence MacCarthy, Santigie Sesay

**Affiliations:** 1 Helen Keller International, Freetown, Sierra Leone, 35 Nelson Lane, Tengbeh Town, Freetown, Sierra Leone; 2 National Neglected Tropical Disease Control Program, Ministry of Health and Sanitation, New England, Freetown, Sierra Leone

**Keywords:** Lymphatic filariasis, Monitoring and evaluation, Mass drug administration, Community health workers, Supply chain management, Urbanization

## Abstract

**Background:**

Since 2007 Sierra Leone has conducted mass drug administration (MDA) for the elimination of lymphatic filariasis (LF) implemented by unpaid community health volunteers (CHVs). Other health campaigns such as Mother and Child Health Weeks (MCHW) pay for services to be implemented at community level and these persons are then known as community health workers (CHWs). In 2010, the LF MDA in the 12 districts of the Southern, Northern and Eastern Provinces un-expectantly coincided with universal distribution of Long Lasting Insecticide Treated Nets (LLITNs) during the MCHW. In-process monitoring of LF MDA was performed to ensure effective coverage was attained in hard to reach sites (HTR) in both urban and rural locations where vulnerable populations reside.

**Methods:**

Independent monitors interviewed individuals eligible for LF MDA and tallied those who recalled having taken ivermectin and albendazole, calculated program coverage and reported results daily by phone. Monitoring of coverage in HTR sites in the 4 most rapidly urbanizing towns was performed after 4 weeks of LF MDA and again after 8 weeks throughout all 12 districts. End process monitoring was performed in randomly selected HTR sites not previously sampled throughout all 12 districts and compared to coverage calculated from the pre-MDA census and reported treatments.

**Results:**

Only one town had reached effective program coverage (≥80%) after 4 weeks following which CHWs were recruited for LF MDA in all district headquarter towns. After 8 weeks only 4 of 12 districts had reached effective coverage so LF MDA was extended for a further month in all districts. By 12 weeks effective program coverage had been reached in all districts except Port Loko and there was no significant difference between those interviewed in communities versus households or by sex. Effective epidemiological coverage (≥65%) was reported in all districts and overall was significantly higher in males versus females.

**Conclusions:**

The challenges to LF MDA included the late delivery in country of ivermectin, the availability and motivation of unpaid CHVs, concurrent LLITN distribution and the MCHW, remuneration for CHWs, rapid urbanization and employment seeking population migrations. 'In process' monitoring ensured modifications of LF MDA were made in a timely manner to ensure effective coverage was finally attained even in HTR locations.

## Background

Lymphatic filariasis (LF) is a mosquito borne neglected tropical disease (NTD) [1]. In 1998, the World Health Organization (WHO) launched a Global Program for the Elimination of LF [2]. Persons suffering from LF may suffer morbidity, social marginalization and loss of wage-earning capacity. As LF is a disease of the most vulnerable they often live in remote, under-populated, rural, hard to reach (HTR) locations, or in over-populated, rapidly urbanizing settlements increasingly recognized as HTR by persistently underperforming in MDAs even though they are geographically accessible. Ensuring effective coverage for HTR populations should be prioritized in LF elimination programs.

Mapping of LF in Sierra Leone was performed in 2005 and all 14 districts were found to be endemic, justifying nation-wide mass drug administration (MDA) and a baseline LF microfilaria survey was performed in 2007–08 [3]. The goal of the National Neglected Tropical Disease Control Program (NTDCP) is to eliminate LF by 2015, requiring 5–6 effective rounds with ivermectin (IVM) plus albendazole (ALB) [4]. To be effective, 65% of the national, at risk population should receive IVM and ALB or 80% of the eligible, at risk population [5]. Children <90 cm in height, the sick, very elderly, pregnant women, and women who gave birth within the last week are not eligible during MDA [6].

**Figure 1:**
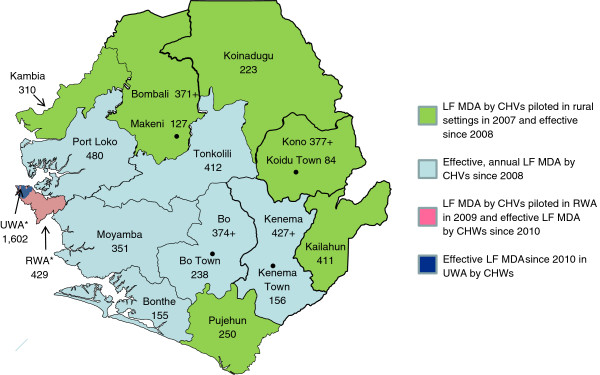
Phases of scale up of LF MDA in Sierra Leone, illustrating pre-MDA census data (*1000) for 2010 by district and the 4 largest towns

The National NTDCP piloted house-to-house LF MDA by unpaid community health volunteers (CHV) in 6 districts in 2007 [7]. Since 2008 all 12 districts in the Northern, Southern and Eastern Provinces have implemented effective MDA using CHVs as reported by the NTDCP. However a post event coverage survey found house-to-house LF MDA by CHVs had been ineffectual in the semi-urban setting of the Rural Western Area (RWA) in 2009 [8]. The LF MDA strategy was changed to a public, fixed point and street-by-street distribution using temporary paid community health workers (CHWs) in the RWA and Urban WA (UWA) in June 2010 and end process independent monitoring showed that coverage had been effective as shown in Figure 1[9]. Full geographical coverage including the 2 districts in the Western Area was achieved in 2010 [9,10].

Unpaid CHVs are elected/re-elected annually by their communities, trained by the Peripheral Health Unit (PHU) staff and function as the drug distributors for LF MDA. These CHVs perform an annual pre-MDA community census, but un-controlled settlement of internally displaced persons during the war (1991–2002) followed by employment seeking migrations post war makes this pre-MDA census challenging in rapidly urbanizing settings [8]. Trained CHVs may also work for and receive remuneration as CHWs from other programs such as the National Malaria Control Program (NMCP) and/or twice annually from the Mother and Child Health Week (MCHW). Unpaid CHVs and temporary, paid CHWs are increasingly implementing health programs in Sierra Leone.

The health calendar became 'over-booked' in 2010. Delayed LF MDA coincided with the MCHW, which in addition to mass Vitamin A supplementation (VAS), de-worming and polio immunization to pre-school children, undertook universal distribution of Long Lasting Insecticide Treated Nets (LLITNs).

If LF MDAs do not reach effective coverage, programs may need to be continued [11,12]. The NTDCP and implementing partner: Helen Keller International (HKI) were aware of the negative impact that the concurrent MCHW and LLITN distribution could have on coverage especially in HTR communities. The most rapidly urbanizing, largest towns with relatively large HTR populations include the 3 provincial head quarters of the North, South and East: Makeni, Bo and Kenema respectively. These towns had grown rapidly during the war and post war due internal displacement. In addition Koidu in the heart of the Kono diamond mining district has grown most rapidly post war due to employment seeking migration. In-process monitoring of LF MDA was performed with the specific objectives of ensuring effective coverage was attained in HTR sites, enabling modifications/re-enforcements to the MDA strategy before the distribution stopped. The main objective of the end process monitoring was to compare program coverage in HTR locations by district and the 4 largest towns with epidemiological coverage reported by the District Health Management Teams (DHMTs) to the NTDCP calculated from the pre-MDA census and reported treatments. The second objective was to compare coverage in males versus females by both methods. This paper reports on the results, discusses the challenges to effective LF MDA and the corrective measures taken.

## Methods

### Pre-Mass Drug Administration census

Preparations began 2 months before for the annual MDA in 2010 with advocacy meetings and social mobilization organized by the staff of the 1,080 local PHUs and their community health management committees in 12 districts comprised of 149 chiefdoms and approximately 14,253 communities. Each PHU has a recognized catchment population of approximately 5,000 residents. These communities elected or re-elected approximately 2 CHVs per 500 residents. These CHVs then up-dated the pre-MDA community census upon which IVM and ALB requisitions from their supervisory PHUs were based and reported epidemiological coverage was finally calculated.

### Mass Drug Administration

The DHMT compiled all the population data from their district and the LF MDA was scheduled for 8 weeks to be completed before the MCHW and LLITN distribution. Due to the late arrival of IVM in country, LF MDA was delayed and coincided with the MCHW and LLITN distribution by which time many CHVs had been recruited as paid as CHWs. Distribution of LLITNs was prioritized in many communities due to this remuneration as opposed to the unpaid IVM /ALB distribution. Supportive supervision was performed by PHU staff who in turn were supervised by the DHMTs. Monitoring of the 12 DHMTs was performed by the NTDCP.

### Independent Monitors, training tools and protocol

Independent monitoring tools and protocols had been developed for LF MDA in June 2010 for the Western Area by HKI [8]. These tally sheets were modified for the 12 districts with the addition of ‘court barries’ the traditional rural meeting points. Monitors were trained then assigned to a district where they could speak at least 2 of the local languages. Monitors recorded the number of persons interviewed each day and of those who recalled having taken IVM and ALB during the current LF MDA. Monitors first interviewed people in various busy community locations (court barries, markets, bus stops, churches/mosques, school environs) and in households. Then household interviews were performed on the fringes of the nearest PHUs’ catchment area. In the Sierra Leone context it would be unlikely to find a respondent in both a central location and within a household on the fringes of the catchment area within the hours that the monitoring was performed. The monitors were trained and aware that no duplication of interviews were to be recorded.

The geographical size of a catchment area of a PHU varies dependent upon the population density it serves. The 'fringes' was estimated by the monitor by a 10 minute walk away from the nearest PHU in an urban setting and a 20 minute walk in a rural setting, without entering the catchment area of an adjacent PHU. At that point the monitor selected the nearest household and started the sampling, continuing with the adjacent household until 30 households had been sampled. Within a household one eligible person was randomly selected by the distribution of numbers written on folded paper, selection of a number by a household member, then the person with that number was interviewed and their response recorded. Monitors reported results daily by phone to the HKI coordinator who forwarded the compiled data to the NTDCP for further dissemination and action by DHMTs.

### In process monitoring: sample size, sites and selection

The HTR sites were purposely selected from a list of enumeration areas (EAs) identified by the WHO/MoH as persistently underperforming in past MDAs for VAS and/or the 5 rounds of polio immunization in 2010. No additional resources had been made available to these HTR sites in the interim. 'In process' monitoring was conducted after 4 weeks of LF MDA in HTR sites in the 4 most rapidly urbanizing towns: Makeni, Koidu, Bo and Kenema. A minimum of 180 interviews (30 from each community location and 30 within households) from each site were performed in each of 45 HTR sites: Makeni: 11, Bo 14, Kenema: 12 and Koidu 8, proportionate to their estimated HTR populations.

Monitoring was repeated after 8 weeks of LF MDA in all 12 districts. As effective coverage had been reached in Koidu town at 4 weeks it was not monitored again at 8 weeks. Monitors visited 3 HTR sites, randomly selected from randomly selected chiefdoms within their assigned district and HTR sites in 3 provincial head quarter towns. A minimum of 180 interviews were performed in each of 45 sites (3 chiefdoms per district plus 3 in each of the provincial head quarter towns).

### End process monitoring and program coverage

**Figure 2:**
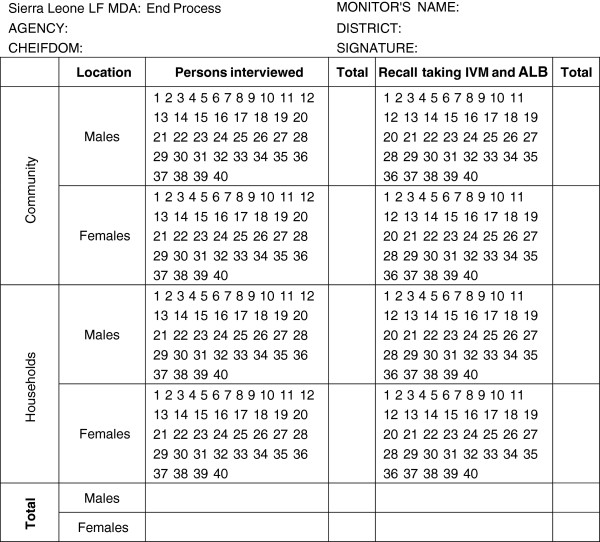
Independent monitors tally sheet used at 'end process' disaggregated by sex for community and household interviews

'End process' monitoring was conducted after 12 weeks of LF MDA in 3 randomly selected HTR sites, within chiefdoms not previously monitored within all 12 districts and the 3 provincial head quarter towns: 45 sites. At end process the tally sheets information was disaggregated by sex as shown in Figure 2: a minimum of 30 males and 30 females were interviewed in both community and household locations per site.

### Epidemiological coverage from NTDCP reported treatments and pre MDA census

The CHVs reported those treated, disaggregated by sex to their supervisory PHU staff. These were compiled and forwarded to the DHMTs. The NTDCP collected the district coverage data within 2 weeks of the cessation of LF MDA and followed up on any un-reported PHUs before compiling the national figures which are then reported to the Ministry of Health and Sanitation (MoHS) and partners: WHO, Sightsavers and HKI.

### Data management and statistical analysis

The data from the in and end process independent monitoring was recorded on tally sheets and reported by phone daily to the coordinator. Data was entered into excel and coverage results calculated and disseminated to the NTDCP and onwards to the DHMTs for further action. After each round of independent monitoring the monitors were 'debriefed' by the coordinator and the NTDCP so that individual reports of best practice and challenges could be compiled and lessons learnt further disseminated. The end process independent monitoring data was compared to the NTDCP reported treatments and pre-MDA census, analyzed by Chi-squared (χ2) test to determine significant difference between sexes, household and communal location at 5% level of significance.

### Ethical considerations

No compensation or other incentive was offered to respondents. To protect the confidentiality of information shared with the monitors, no names or addresses were recorded. The monitoring was approved by the Sierra Leone Ministry of Health and Sanitation responsible for medical research and ethics.

## Results

### In process coverage in HTR sites in large towns after 4 weeks and district-wide after 8 weeks of LF MDA

After 4 weeks of LF MDA, a total of 8,193 persons were interviewed in the 4 towns and 65.3% recalled taking both IVM and ALB as shown in Table 1. Only Koidu had reached effective coverage after 4 weeks of LF MDA. Following this report CHWs were recruited and paid $2 a day for 5 days of LF MDA in all 12 district head quarter towns.

**Table 1:** Program coverage by largest towns and district: In process and End process disaggregated by sex

	**In process after 4 weeks of LF MDA HTR sites in large towns only**			**In process after 8 weeks of LF MDA in HTR sites**			**End Process After 12 weeks of LF MDA disaggregated by sex in HTR sites**						**End Process: Total**		
**Location**	**N**	**Recall**	**%**	**N**	**Recall**	**%**	**Males**	**Recall**	**%**	**Females**	**Recall**	**%**	**N**	**Recall**	**%**
**SOUTHERN PROVINCE**															
Bo Town*	2,505	1,914	76.4	610	530	86.9	398	370	93.0	418	404	96.7	816	774	94.9
Bo District				750	490	65.3	439	408	92.9	442	387	87.6	881	795	90.2
Bonthe				710	360	50.7	405	363	89.6	398	354	88.9	803	717	89.3
Moyamba				702	569	81.1	402	329	81.8	404	331	81.9	806	660	81.9
Pujehun				750	576	76.8	329	274	83.3	330	278	84.2	659	552	83.8
**EASTERN PROVINCE**															
Kenema Town*	2,185	1,274	58.3	775	621	80.1	283	226	79.9	300	237	79.0	583	463	79.4
Kenema District				606	419	69.1	359	325	90.5	371	338	91.1	730	663	90.8
Kailahun				692	606	87.6	373	316	84.7	392	326	83.2	765	642	83.9
Koidu/Kono	1,475	1,179	79.9	730	660	90.4	370	339	91.6	370	343	92.7	740	682	92.2
**NORTHERN PROVINCE**															
Makeni*	2,028	984	48.5	567	471	83.1	413	351	85.0	407	356	87.5	820	707	86.2
Bombali				750	517	68.9	442	377	85.3	432	336	77.8	874	713	81.6
Tonkolili				750	532	70.9	360	297	82.5	349	291	83.4	709	588	82.9
Koinadugu				727	501	68.9	450	370	82.2	450	369	82.0	900	739	82.1
Port Loko				750	492	65.6	420	275	65.5	420	302	71.9	840	577	68.7
Kambia				666	537	80.6	448	374	83.5	450	365	81.1	898	739	82.3
**Overall**	8,193	5,351	65.3	10,535	7,881	74.8	5,891	4,994	84.8	5,933	5,017	84.6	11,824	10,011	84.7

No statistical significant difference by household versus community location in each phase or by sex at end process.

* Provincial Head Quarter Towns.

After 8 weeks of LF MDA, a total of 10,535 persons were interviewed and 74.8% recalled taking both IVM and ALB were (Table 1). As Koidu had already achieved effective coverage it was not monitored again at 8 weeks. The remaining 3 large towns but only 4 of 12 districts had reached effective program coverage at 8 weeks: Moyamba, Kailahun, Kono and Kambia. Overall there was no significant difference in coverage in those interviewed in the community: 75.5%, versus households: 71.3%. Following this report LF MDA was extended for a further month in all 12 districts.

### End process program coverage in HTR sites

After 12 weeks of LF MDA, a total of 11,824 persons were interviewed in the 12 districts and 4 large towns and 84.7% recalled taking both IVM and ALB (Table 1). Only Port Loko had not reached effective program coverage at 68.7%. Overall, there was no significant difference in coverage by those interviewed in the community versus households or by sex. The greatest difference in coverage between the sexes was in Port Loko males: 65.5%, versus females: 71.9%, but this was not statistically significant.

### Epidemiological coverage from NTDCP treatment reports and the pre MDA census

The total population in the 12 districts was 4,749,557, males: 48%, females: 52% as shown in Table 2. A total of 3,571,514 treatments of IVM and ALB had been given and overall epidemiological coverage was 75.2%. All districts had reached the effective coverage. Overall, there was significantly higher coverage in males versus females (p<0.0001). In Bo town and in 5 districts there was significantly greater coverage in males versus females (p<0.05, p<0.0001 respectively). In Kenema town and in 5 districts there was significantly greater coverage in females versus males (p<0.0001).

**Table 2:** Epidemiological coverage from reported treatment tallies and the pre-MDA census and by district, large towns and sex

**District and large towns**	**Pre-MDA census by sex**			**Population treated and coverage by sex**				**Overage coverage**		**Coverage in males versus females**
	**Males**	**Females**	**Total**	**Males**	**Females**	**Males**	**Females**	**Total treated**	**%**	**p value**
**SOUTHERN PROVINCE**										
Bo Town*	118,992	119,447	238,439	84,859	81,169	71.3%	68.0%	166,028	69.6%	p<0.0001
Bo	183,454	191,284	374,738	137,869	142,098	75.2%	74.3%	279,968	74.7%	p<0.005
Bo Town	118,992	119,447	238,439	84,859	81,169	71.3%	68.0%	166,028	69.6%	p<0.0001
Bonthe	74,459	80,401	154,860	54,065	63,136	72.6%	78.5%	117,201	75.7%	p<0.0001
Moyamba	166,492	184,287	350,779	144,942	123,934	87.1%	67.3%	268,876	76.7%	p<0.0001
Pujehun	121,747	128,533	250,280	94,862	98,623	77.9%	76.7%	193,485	77.3%	p<0.0001
**EASTERN PROVINCE**										
Kenema Town*	77,345	78,567	155,912	56,661	59,139	73.3%	75.3%	115,800	74.3%	p<0.0001
Kenema	208,519	218,847	427,366	162,680	171,283	78.0%	78.3%	333,963	78.1%	NS
Kailahun	191,421	219,088	410,509	148,492	173,713	77.6%	79.3%	322,206	78.5%	p<0.0001
Kono	184,986	192,090	377,076	136,155	147,284	73.6%	76.7%	346,719	91.9%	p<0.0001
Koidu Town	43,126	41,360	84,486	31,500	31,780	73.0%	76.8%	63,280	74.9%	p<0.0001
**NORTHERN PROVINCE**										
Makeni Town*	63,444	63,556	127,000	44,477	44,831	70.1%	70.5%	89,308	70.3%	NS
Bombali	173,271	197,844	371,115	127,444	146,326	73.6%	74.0%	273,770	73.8%	NS
Tonkolili	197,723	214,682	412,404	138,908	165,287	70.3%	77.0%	304,195	73.8%	p<0.0001
Koinadugu	106,047	116,920	222,966	75,271	86,788	71.0%	74.2%	162,059	72.7%	p<0.0001
Port Loko	231,117	249,803	480,920	177,152	185,875	76.7%	74.4%	363,026	75.5%	p<0.0001
Kambia	146,617	164,088	310,705	113,026	121,884	77.1%	74.3%	234,910	75.6%	p<0.0001
**Overall**	2,288,760	2,460,797	4,749,557	1,728,363	1,843,150	75.5%	74.9%	3,571,514	75.2%	p<0.0001

NS=Not significant.

### Corrective measures taken to ensure effective LF MDA

The concurrent LF MDA, MCHW and LLITN distribution placed enormous work load on the DHMTs-monitoring systems with limited human and logistical resources. The additional support given by independent monitors was found invaluable. Independent data on low coverage enabled the DHMTs to focus supportive supervision on the PHU staff and CHVs in these sites. Additional qualitative information by the monitors regarding stock distribution, poor cooperation by staff, community members or leaders, could also be rapidly addressed by the DHMTs. The independent monitors were also able to directly address concerns or misunderstandings regarding LF MDA by individuals, communities and staff in these sites or refer them to the PHU staff/DHMT.

The 4 largest towns were purposefully selected for monitoring after weeks of LF MDA as it is recognized that distribution by CHVs in rapidly urbanizing settings in Sierra Leone is weak [8]. Lack of clear demarcation of PHU catchment areas, lack of community 'identity' in these new settlements and of community health committees makes the election and retention of urban CHVs difficult. The low coverage at 4 weeks resulted in the mobilizations of paid CHWs for LF MDA in all 12 district head quarter towns by the NTDCP.

After 8 weeks when LF MDA had been due to cease, monitoring found that all 4 large towns but only 4 districts had reached effective coverage which justified the extension of LF MDA for a further 4 weeks. Without the independent monitoring the NTDCP would not have had coverage reports until LF MDA had ceased as scheduled after 8 weeks and the DHMT-tallies compiled.

## Discussion

In Koidu the distribution of IVM/ALB and LLITNs had been successfully integrated under the initiative of the DHMT and effective program coverage achieved within 4 weeks of LF MDA. Makeni which had borne the greatest work-load with the presidential launch of the MCHW-LLITN had the lowest IVM/ALB coverage at 4 weeks. In the Dominican Republic the challenges of integrating the LF program into primary health care demonstrated the importance of engaging senior management at an early stage.[13] Collaboration between NMCP and NTDCP-managers could have integrated LLITN and LF MDA distribution as in Central Nigeria [14].

### Comparison of program coverage by independent monitors and epidemiological coverage from CDD reports and pre-MDA census

Effective coverage measured by program coverage of eligible population versus epidemiological coverage of the entire population is based upon the estimate that 15% of the population is ineligible for MDA [15]. Currently in Sierra Leone 17.8% the population are estimated to be ineligible as they are under-five years of age (<90cms tall) [16]. As maternal mortality is very high in Sierra Leone the period of ineligibility for recently delivered women has been extended by the National NTDCP to 2 weeks, making an estimated further 2.4% of the population ineligible [17]. A further 1.6% is estimated ineligible due to sickness giving a higher total of those ineligible for MDA of 21.8% [18]. All districts reported effective coverage by end process by both methods of assessment with the exception of Port Loko where program coverage was 68.5% versus epidemiological coverage of 75.5%.

### Competitive community health 'market' and Process Indicators

Volunteering as an unpaid CHV requires an individual’s commitment and the timing of MDA needs to be compatible with their other personal and professional obligations: family, cultural festivities, teaching, farming, or payment opportunities as a CHW. The seasonal availability of this 'army' of CHVs upon which NTD programs are built should be recognized as a key ‘Process Indicator’ of equal importance as funding, logistics, policy, guidelines, supplies and training [5]. The NTDCP had trained 29,742 CHVs to implement LF MDA. The MCHW then recruited 17,700 CHWs and 8,850 LLITN distributors all drawn from the same community health sector. The work delegated to communities by various programs in countries such as Sierra Leone can over-load their capacity. During this MCHW, over a 3 million doses of Vitamin A, albendazole and/or oral polio vaccinations were given and 2.4 million LLITNs were distributed [19].

### Motivation and remunerations of CHVs and CHWs

In 2009 LF MDA coverage in the Western Area was found to be 22.9% in a post event survey performed within 3 months [8]. Program managers and the DHMT-WA felt that volunteerism did not work well in over-populated, rapidly urbanizing, mixed ethnic communities. As a result in 2010 and 2011 CHWs were paid $3 a day for 5 days LF MDA and independent monitoring immediately at the 'end process' found program coverage of 85.8% and 78.9% respectively [9,20].

The NTDCP and partners have been providing non-cash incentives to CHVs such as T-shirts and certificates and encourage community leaders to provide incentives such as exemption from community levies. The cost of LF MDA would be unaffordable if all CHVs received cash remuneration from the NTDCP [21,22]. This lack of remuneration is increasingly problematic as other programs offering cash remuneration of $2-5 a day scale up. Ensuring CHVs remain motivated is essential for the remaining 5th and 6th round of LF MDA and will require tailored communication messages for community leaders and the public. The MoHS is increasingly aware of the deployment, sometimes conflicting obligations and disparity of remuneration of CHVs/CHWs. A policy has recently been drafted and under review for making provision for standardized remuneration to CHWs who would be trained to provide health care and distribute nutrition, hygiene and sanitation messages at the community level.

### Rapid urbanization: Employment-seeking migration between districts

When there is a marked difference in coverage by reported treatments versus monitored results one explanation is that a greater number of persons took the drugs than were expected based upon those enumerated in the pre-MDA census [5]. The building of a railway through Port Loko and a marine terminal for the exportation of iron ore resulted in sudden employment opportunities in mid 2010 and may account for the disparity observed. It was noted that in some districts a few MoHS staff had also recently left their posts for employment by these new, iron-ore companies. A similar disparity between monitored and reported coverage has been observed since 2009, in Kono and Moyamba where employment opportunities in the mining sector resumed for diamonds/gold and rutile post war [10].

It is generally expected that a higher percentage of males access MDA than females due to uncertainty related to pregnancy status. This was the case overall and in 5 districts but not so in another 5 districts. This could also be due to male employment-seeking emigration making less males available for treatment than had been enumerated. The higher percentage of males enumerated in Koidu (51%) may also be due to the mining opportunities there.

The pre-MDA census is recognized by NTDCP and HKI to be weak in rapidly urbanizing settings. Employment opportunities in the iron-ore mining sector, bio-energy production and support industries in Bombali and Tonkolili in 2011 have also created migrations which could affect program coverage unless innovative strategies for LF MDA are employed: going to work sites to treat employees and/or the collaboration with the employers medical teams, labour and employment agencies. This is a move away from the village MDA by CHVs under the control of the PHU staff and as migrations are occurring rapidly, these new strategies need to be developed for the 5th and 6th round to ensure effective coverage is maintained in preparation for stopping LF MDA. In addition cross border migration into Sierra Leone for MDAs from neighboring Guinea and Liberia has become increasingly recognized by many DHMTs.

### Supply chain management

In process monitoring reports also justified the re-distribution of IVM and ALB within districts by DHMTs where populations exceeded expectations based upon the pre-MDA census. Every year after LF MDA the NTDCP submit their reported coverage to the WHO and partners including utilization, wastage, returned drugs, stock balance and projected target population for the subsequent round. The WHO review the request, coordinate supplies and schedules for shipment in readiness for the following round. In 2010 the 1st round of MDA in the Western Area had been planned for January but the WHO-led regional 4 rounds of polio immunization were prioritized and LF MDA had been postponed to June. Consequently the documentation of stock balances and the delivery of the IVM was delayed.

### Cost implications

The cost of performing 'in process' and 'end process' monitoring for LF MDA in these 12 districts in 2010 was $15,994. This represented an additional 11.3% cost of the MDA implementation budget excluding drugs, technical support from implementing partners, GoSL salaries and infrastructure. The limited objectives of the study (to ensure effective coverage was attained and compare survey with reported coverage) enabled this to be performed in relatively few sites at an affordable cost. To expand the study to enable statistical analysis between districts would have increased the cost to over $60,000, beyond the scope of the NTD budget [23]. The cost of recruiting CHWs in the 12 towns for 5 days distribution of IVM and ALB was $5,122.

### Study limitations

Random selection of sites from a list of HTR sites in preference to proportional population size sampling (PPS) or Lot Quality Assurance Survey (LQAS) was implemented to ensure vulnerable populations were specifically sampled [24,25]. In rapidly urbanizing settings the pre-MDA census may be inaccurate. Under-performing/HTR sites identified from the recent polio campaigns represent a mixture of geographically remote, under-populated villages and new, often densely populated settlements. Under-performing HTR sites may change rapidly even within a few months due to PHU staff and/or CDD re-assignment, attrition and/or population migrations.

## Conclusions

Effective coverage of LF MDA was facilitated by purposeful 'in process' monitoring of urban and rural HTR sites, recruitment of additional CHWs in urban locations and a four week implementation extension. By the end process, effective program coverage had been achieved in 11 of 12 districts and there was no statistically significant difference found between those interviewed in central community locations versus households on the fringes of a health centers' catchment area or by sex. Overall, there was a significantly higher reported epidemiological coverage in males versus females. Employment seeking migration in Port Loko could explain low end process program coverage in comparison to the reported epidemiological coverage. Modifications to LF MDA in other rapidly urbanizing districts: Tonkolili and Bombali for LF MDA rounds 5 and 6 are recommended. Integration of LLITN distribution and LF MDA was initiated in Koidu town successfully. Challenges of cross border and rapid internal migration require pro-active planning for subsequent MDAs to maintain effective coverage in vulnerable populations. The additional cost of 'in' and 'end process' monitoring to ensure effective coverage are justified in comparisons to the cost of additional rounds of MDA that may be required if effective coverage is not achieved.

## Competing interests

The authors state that there are no conflicts of interest.

## Authors’ contributions

MH designed and conducted the study. MH and MS recruited and trained the independent monitors and compiled their data. SS, AC and FM managed the pre-MDA census, MDA and post-MDA data compilation. MH analyzed and interpreted the data. HT and MS drafted and MH revised the paper. All authors reviewed and approved the final manuscript.

## Disclaimer

This study is made possible by the generous support of the American people through the United States Agency for International Development (USAID). The contents are the responsibility of the authors and do not necessarily reflect the views of USAID or the United States Government.

## Funding

This survey was funded by the USAID NTD Control Program through a grant to Helen Keller International under a cooperative agreement with RTI International.
